# Awareness of Evidence-Based Treatments Among Women with Dyspareunia: A Cross-Sectional Survey Study

**DOI:** 10.3390/jcm15093408

**Published:** 2026-04-29

**Authors:** Wiktoria Sztandera, Anita Ewa Sikora-Szubert, Karolina Zajdel, Radosław Zajdel, Robert Irzmański

**Affiliations:** 1 Department of Internal Medicine, Rehabilitation and Physical Medicine, Medical University of Lodz, 90-647 Lodz, Poland; wiktoria.sztandera@umed.lodz.pl; 2 Department of Pathology of Pregnancy and Gynecology, Medical University of Lodz, 92-213 Lodz, Poland; anita.sikora-szubert@umed.lodz.pl; 3 Department of Public Health, Medical University of Lodz, 90-752 Lodz, Poland; karolina.smigiel@umed.lodz.pl; 4 Department of Economic and Medical Informatics, University of Lodz, 90-214 Lodz, Poland; 5 Department of Internal Diseases, Rehabilitation and Physical Medicine, Medical University of Lodz, 90-647 Lodz, Poland; robert.irzmanski@umed.lodz.pl

**Keywords:** dyspareunia, pelvic floor physiotherapy, patient awareness, sexual pain disorder, health education

## Abstract

**Background:** Dyspareunia is a common female sexual pain disorder that significantly impairs quality of life. Despite the availability of evidence-based treatments, including multimodal pelvic floor physiotherapy and psychosexual interventions, patient awareness of these options remains insufficiently characterized. This study aimed to assess knowledge of dyspareunia management among affected women and to identify independent predictors of awareness. **Methods:** A cross-sectional survey was conducted in 2023 at the Central Clinical Hospital of the Medical University of Lodz, Poland, among 72 women with physician-confirmed dyspareunia. An 82-item questionnaire administered via structured face-to-face interviews assessed sociodemographic characteristics, clinical features including intercourse positions, penetration depth, and partner-related factors, and knowledge of pelvic floor therapy. Responses to 18 knowledge items were aggregated into a synthetic awareness variable (range 0–24 points). Internal consistency was evaluated using Cronbach’s alpha. Statistical analysis included item-level scoring, multiple linear regression, Mann–Whitney U test, Kruskal–Wallis test, and Spearman’s rank correlation. Effect sizes are reported as Cohen’s d for parametric comparisons and rank-biserial correlation for nonparametric comparisons. **Results:** The mean awareness score was 10.9 ± 6.1 out of 24 points. The awareness scale demonstrated good internal consistency (standardized Cronbach’s α = 0.880). Item-level analysis revealed critical knowledge gaps: biofeedback was recognized by only 15.3% of participants, and only 6.2% could correctly estimate the number of pelvic floor muscles. In multiple linear regression (R^2^ = 0.224, adjusted R^2^ = 0.153, *p* = 0.009), age (β = −0.305, *p* = 0.009) and current urogynecological physiotherapy use (β = 0.332, *p* = 0.019) were independent predictors of awareness. Physiotherapy users scored on average 5.6 points higher than non-users (16.0 ± 4.9 vs. 10.4 ± 6.0; *p* = 0.027; rank-biserial r = 0.51), although this finding should be interpreted with caution given the small number of physiotherapy users (*n* = 7) and the wide confidence interval. More than half of participants (55.6%) reported positional dependency of dyspareunia; in exploratory analyses, none of the assessed dyspareunia characteristics showed a statistically significant association with awareness. Younger women (≤24 years) demonstrated significantly higher awareness than older participants (12.1 ± 5.6 vs. 9.1 ± 6.5; *p* = 0.039; Cohen’s d = 0.51). **Conclusions:** Women with dyspareunia demonstrate modest and heterogeneous awareness of evidence-based treatments, with the largest deficits in knowledge of specific physiotherapeutic modalities. These findings highlight the need for targeted educational interventions and improved referral pathways to pelvic floor physiotherapy. This study establishes a conceptual framework for assessing patient awareness of dyspareunia treatments, which warrants validation in larger, multi-center studies.

## 1. Introduction

Dyspareunia—persistent or recurrent pain associated with sexual intercourse—is a common female sexual pain disorder, with prevalence estimates commonly ranging from 3% to 18% and lifetime prevalence reported at approximately 10% to 28% [1,2]. Contemporary classifications have moved away from the older organic/psychogenic dichotomy toward a biopsychosocial understanding of sexual pain disorders. In ICD-11, sexual pain related to penetration is classified under sexual pain-penetration disorder (HA20), a concept that reflects the integration of somatic, psychological, and relational determinants. This reconceptualization has important clinical implications, emphasizing that effective management should address the interplay of peripheral pain mechanisms, central sensitization, pelvic-floor dysfunction, and psychosexual factors [3,4].

From a clinical standpoint, dyspareunia is best approached as a heterogeneous syndrome with multiple underlying causes rather than a single uniform disorder [5]. Superficial pain, exemplified by provoked vestibulodynia, is associated with increased intraepithelial innervation and peripheral sensitization within the vulvar vestibule [6]. By contrast, deep dyspareunia is more commonly associated with conditions such as endometriosis, pelvic adhesions, pelvic inflammatory disease, adnexal pathology, or pelvic congestion [5].

Adenomyosis—defined by the presence of endometrial glands and stroma within the myometrium—should be recognized as a distinct and clinically relevant cause of dyspareunia. In a retrospective cohort of 307 women with adenomyosis, dyspareunia was reported by 59.2% of those with concurrent endometriosis and 46.3% of those with adenomyosis alone [7]. Furthermore, adenomyosis may diminish the effectiveness of surgical treatment for dyspareunia in patients with rectovaginal septum endometriosis: women with coexistent adenomyosis demonstrated significantly less improvement in pain scores and sexual quality of life after surgery compared to those with isolated endometriosis [8]. These findings support the conceptualization of adenomyosis as a distinct clinical entity contributing independently to sexual pain, rather than merely a phenotype of endometriosis.

Pelvic congestion syndrome (PCS) is another often-underdiagnosed condition estimated to account for up to 30% of chronic pelvic pain cases. Dyspareunia—particularly postcoital dyspareunia—is a leading symptom of PCS, and the specific type of dyspareunia may help clinicians differentiate between PCS and endometriosis at initial assessment: while endometriosis-related dyspareunia is typically deep, positional, and cyclical, PCS-associated dyspareunia tends to present as a dull, non-cyclical ache that worsens after intercourse [9].

Postpartum dyspareunia represents another clinically important presentation, often associated with obstetric intervention and perineal trauma [10]. Such clinical subclassification can guide diagnostic evaluation and inform management decisions [5], and underscores the need for patients to be adequately informed about the nature of their condition and the range of available therapeutic options.

A growing body of evidence supports several first-line or early conservative interventions for specific dyspareunia subtypes. Multimodal pelvic-floor physical therapy (PFPT)—combining education, pelvic-floor muscle training with biofeedback, manual techniques, dilator work, and home exercises—has been shown in a multicenter randomized trial to reduce intercourse pain more effectively than topical lidocaine, with benefits sustained at six months [11]. Cognitive-behavioral couple therapy (CBCT) has likewise shown superiority to lidocaine on several psychosexual outcomes in women with provoked vestibulodynia, including pain unpleasantness at follow-up, sexual distress, treatment satisfaction, and global sexuality improvement [12,13]. Complementary components such as breathing and relaxation training are commonly incorporated into contemporary PFPT programs, consistent with evidence of functional co-activation between the pelvic floor and the surrounding abdominal-pelvic musculature [14]. For Genitourinary Syndrome of Menopause (GSM)-related dyspareunia, systematic review evidence suggests that vaginal estrogens may improve symptoms, while intravaginal prasterone and oral ospemifene also show benefit for dyspareunia and related outcomes [15]. For endometriosis-associated pain, oral gonadotropin-releasing hormone (GnRH) antagonists with hormonal add-back therapy provide meaningful symptom control, particularly for dysmenorrhea and non-menstrual pelvic pain [16,17]. Expert reviews and consensus statements support PFPT and psychosexual interventions as key first-line non-invasive strategies for vulvodynia and provoked vestibulodynia, with pharmacological or surgical approaches typically reserved for selected or refractory cases [18,19].

Despite increasing evidence for several effective management strategies, patient awareness of these options has been only sparsely addressed in the literature. Population-based data from Britain indicate that 7.5% of sexually active women report painful intercourse, while under-recognition, underdiagnosis, and negative clinical experiences remain important barriers to timely care [20,21]. Barriers to care operate at both the patient and clinician level: a 2024 survey of physicians found that only 11.1% routinely screened women for sexual dysfunction, with inadequate training (57.3%) and patient discomfort (60.6%) identified as the most frequent barriers [22]. Survey and review data further suggest that women with chronic vulvar pain often experience delayed diagnosis and misdiagnosis, and that access to evidence-based non-invasive therapies may be inconsistent [21]. However, the extent to which patients with dyspareunia understand the role of the pelvic floor in their symptoms, recognize available therapeutic modalities, or are familiar with the biopsychosocial framework of contemporary management remains insufficiently characterized.

The aim of the present study was therefore to assess the awareness of women with clinically confirmed dyspareunia regarding the etiology of their symptoms and available evidence-based treatments, with particular emphasis on pelvic floor physiotherapy. Additionally, we sought to evaluate the internal consistency of the knowledge assessment instrument, identify item-level knowledge gaps, and determine which sociodemographic and clinical characteristics—including age, education level, pain duration, pain intensity, and physiotherapy use—independently predicted awareness. Identifying specific knowledge deficits may inform the design of targeted educational interventions aimed at reducing diagnostic delay and improving treatment-seeking behavior in this population.

## 2. Materials and Methods

### 2.1. Study Design and Survey Instrument

This cross-sectional survey study was conducted in 2023 at the Central Clinical Hospital of the Medical University of Lodz, Pomorska Street, Lodz, Poland. Women attending gynecological outpatient clinics at the hospital were screened for eligibility by a physician. Eligible participants were women aged ≥17 years with clinically confirmed dyspareunia, diagnosed by a physician based on clinical interview and gynecological examination. Participants were recruited through direct, face-to-face invitation during routine clinical visits. All women who met the eligibility criteria and provided informed consent completed the survey in its entirety via structured face-to-face interview conducted by a physiotherapist; no incomplete responses were recorded and no participants withdrew after consenting. The final sample comprised 72 women. Data were collected using a structured survey method comprising 82 items organized into nine thematic sections (Supplementary Table S1).

The survey began with sociodemographic data including age, educational background, place of residence, marital status, height, and weight. Dyspareunia characteristics were assessed through 15 items covering duration and onset of symptoms, sexual activity frequency, pain intensity assessed using a Visual Analogue Scale (0–10), pain nature and location, situational factors such as intercourse positions and penetration depth, and partner-related factors. Menstrual characteristics included cyclical pain patterns, dysmenorrhea severity and location, and associated symptoms.

Reproductive history comprised questions on pregnancy and parity, mode of delivery, pregnancy complications, perinatal complications including caesarean scar complications, and pain during other perineal activities. The contraception and gynecologic history section addressed current and past hormonal contraception use, frequency of genital infections, and diagnosed gynecologic conditions including erosion, cysts, myomas, endometriosis, polycystic ovary syndrome, and infections, as well as prior gynecologic or pelvic surgeries. Medical history questions covered chronic diseases such as hypertension, diabetes, and autoimmune conditions, along with back pain severity assessed on a Visual Analogue Scale (0–10) and spinal pain location.

Psychological and relational factors were evaluated through questions on stress level, traumatic sexual experiences, emotional responses to first intercourse and current sexual activity, and level of sexual involvement. Treatment history items addressed current and past treatment for sexual complaints, urogynecological physiotherapy utilization, specialist consultations with psychologists, sexologists, or gynecologists, and communication with healthcare providers and partners regarding dyspareunia.

The final section comprised 18 knowledge assessment items evaluating awareness of pelvic floor anatomy, function, and therapeutic modalities. An incorrect answer or “don’t know” response was coded as 0 points, while correct answers were coded from 1 to 3 points depending on question type. The summed point values from these knowledge assessment responses formed an aggregated synthetic variable, defined as “awareness”, ranging from 0 to 24 points. This numerical variable was statistically analyzed in relation to independent variables including age, education level, pain duration, pain intensity, urogynecological physiotherapy use, and treatment history. The complete survey instrument and scoring methodology are presented in Supplementary Tables S1 and S2.

The survey instrument was developed de novo by the author group, as no validated questionnaire assessing patient awareness of evidence-based treatments for dyspareunia was available at the time of study design. Item content was informed by current clinical guidelines and evidence-based literature on pelvic floor physiotherapy, psychosexual interventions, and biopsychosocial management of sexual pain disorders [11,12,14,19]. The 18 knowledge assessment items were constructed to reflect key domains of pelvic floor anatomy, function, and therapeutic modalities that are supported by contemporary evidence. The instrument underwent internal review by the research team (including expertise in gynecology, physiotherapy, and public health) for content relevance, clarity, and comprehensiveness prior to deployment. Internal consistency was subsequently evaluated using Cronbach’s alpha.

It should be noted that the knowledge items were designed to capture a broad spectrum of awareness, ranging from general conceptual understanding (e.g., biopsychosocial underpinnings of sexual dysfunction) to specific anatomical and therapeutic knowledge (e.g., naming pelvic floor muscles, identifying biofeedback). Items assessing detailed anatomical knowledge, such as the number of pelvic floor muscles or naming a constituent muscle, were not included because patients are expected to memorize anatomy, but rather as proxy indicators of prior exposure to structured pelvic floor education or physiotherapy contact. Current physiotherapy use was the strongest positive predictor of awareness in the regression model, consistent with the interpretation that specific anatomical items differentiate women who have received formal education from those who have not.

### 2.2. Statistical Analysis

Descriptive statistics are presented as arithmetic means with standard deviations (SD) for normally distributed variables, and as medians with ranges for non-normally distributed variables. For categorical variables, frequencies and proportions are reported. The normality of quantitative variables was assessed using the Shapiro–Wilk test. Homogeneity of variance was evaluated with Levene’s test.

The internal consistency of the 18-item awareness scale was assessed using Cronbach’s alpha coefficient, calculated for both raw and standardized item scores.

Item-level analysis was conducted by calculating the mean score achieved for each of the 18 knowledge assessment items, expressed as a percentage of the maximum possible score for that item.

For comparisons between two independent groups, Student’s *t*-test was applied when assumptions of normality and homogeneity of variance were met; otherwise, the Mann–Whitney U test was used. For comparisons across three groups, the Kruskal–Wallis test was applied. Effect sizes for parametric group comparisons were quantified using Cohen’s d, with values of 0.2, 0.5, and 0.8 interpreted as small, medium, and large effects, respectively. For the Mann–Whitney U test, the rank-biserial correlation coefficient (r) was calculated as the appropriate nonparametric effect size, with values of 0.1, 0.3, and 0.5 interpreted as small, medium, and large effects.

To identify independent predictors of awareness, a multiple linear regression model was fitted with age, education level, pain duration, pain intensity (VAS), urogynecological physiotherapy use, and current treatment status as predictors. Model fit was assessed using R^2^ and adjusted R^2^. Multicollinearity was evaluated using variance inflation factors (VIF). Standardized regression coefficients (β) are reported alongside unstandardized coefficients (B) with 95% confidence intervals.

Associations between awareness and continuous clinical variables (age, pain intensity, stress level, BMI) were assessed using Spearman’s rank correlation coefficient (rs).

Statistical significance was set at *p* < 0.05 (two-tailed). Statistical analyses were performed using R version 4.5.1 (R Core Team, Vienna, Austria) and Statistica 13.3 (TIBCO Software Inc., Palo Alto, CA, USA).

The primary analysis was the multiple linear regression model, which was specified a priori to identify independent predictors of awareness based on theoretically relevant sociodemographic and clinical variables. Subgroup comparisons (by education level, pain duration, physiotherapy use, and age group) were also predefined to characterize awareness across clinically meaningful strata. The analyses of dyspareunia characteristics in relation to awareness were exploratory and conducted in response to peer review. Given the exploratory nature of several analyses and the small sample size, no formal correction for multiple testing (e.g., Bonferroni) was applied. Consequently, individual *p*-values should be interpreted with caution, and the findings from subgroup and exploratory analyses should be considered hypothesis-generating rather than confirmatory.

## 3. Results

### 3.1. Participant Characteristics

The study included 72 women with confirmed dyspareunia. The cohort was predominantly young (mean age 27.7 ± 9.9 years; 59.7% aged ≤24 years), well-educated (47.2% tertiary, 43.1% secondary education), urban-dwelling (52.8% in cities >100,000 inhabitants), and predominantly single (69.4%). Mean BMI was 24.0 ± 5.0 kg/m^2^. Nearly half of participants (47.2%) reported dyspareunia lasting less than 6 months, while 25.0% had symptoms for more than 2 years. Mean pain intensity was 4.4 ± 2.0 on the VAS (0–10). A comprehensive summary of sociodemographic, clinical, reproductive, and treatment characteristics is presented in Table 1.

### 3.2. Clinical Characteristics and Treatment Utilization

Pain onset patterns varied across the cohort. One-third of participants (*n* = 24, 33.3%) attributed dyspareunia onset to prior genital or urinary tract infections, while 25.0% (*n* = 18) reported pain from sexual debut, 23.6% (*n* = 17) experienced onset following pregnancy or childbirth, and 18.1% (*n* = 13) identified psychological factors as contributors (Table 1). Diagnosed gynecologic conditions were common, with 72.2% (*n* = 52) reporting dysmenorrhea, indicating substantial overlap between dyspareunia and menstrual pain. Only 26.4% (*n* = 19) reported no diagnosed gynecologic condition.

Despite confirmed dyspareunia and diverse underlying etiologies, treatment utilization was strikingly low. Only 12.5% (*n* = 9) were currently receiving any treatment for sexual complaints, and urogynecological physiotherapy was accessed by just 9.7% (*n* = 7). Three-quarters of participants (*n* = 55, 76.4%) had never consulted any specialist regarding their dyspareunia, and 79.2% (*n* = 57) had never discussed it with a gynecologist. Self-directed pelvic floor muscle training had been attempted by 40.3% (*n* = 29), but regular practice (≥3 times per week) was reported by only 8.3% (*n* = 6). These findings highlight a substantial gap between the presence of dyspareunia and engagement with evidence-based therapeutic options, underscoring the clinical relevance of the low awareness scores documented in this cohort.

Dyspareunia characteristics were further detailed with respect to intercourse positions, penetration depth, and partner-related factors. More than half of participants (*n* = 40, 55.6%) reported that pain intensity varied depending on sexual position. Among those reporting positional dependency, the most painful position was the knee-support (doggy style) position (*n* = 15, 37.5%), followed by the missionary position (*n* = 13, 32.5%) and the woman-on-top (cowgirl) position (*n* = 12, 30.0%). The majority of women (*n* = 62, 86.1%) reported that penetration depth did not affect pain intensity, while 13.9% (*n* = 10) indicated that deeper penetration increased pain. Pain most commonly occurred at the onset of intercourse (*n* = 34, 47.2%), followed by pain during intercourse (*n* = 30, 41.7%) and pain at the end of intercourse (*n* = 8, 11.1%).

Regarding partner-related factors, most women (*n* = 53, 73.6%) reported that dyspareunia was not consistent across all sexual partners. Vaginal lubrication was perceived as affecting pain by 51.4% (*n* = 37) of participants. Lubricant use was evenly distributed: 33.3% (*n* = 24) used lubricants regularly, 33.3% (*n* = 24) occasionally, and 33.3% (*n* = 24) never. A history of traumatic sexual experience was reported by 29.2% (*n* = 21) of participants.

### 3.3. Overall Awareness Scores and Internal Consistency

**Figure 1:**
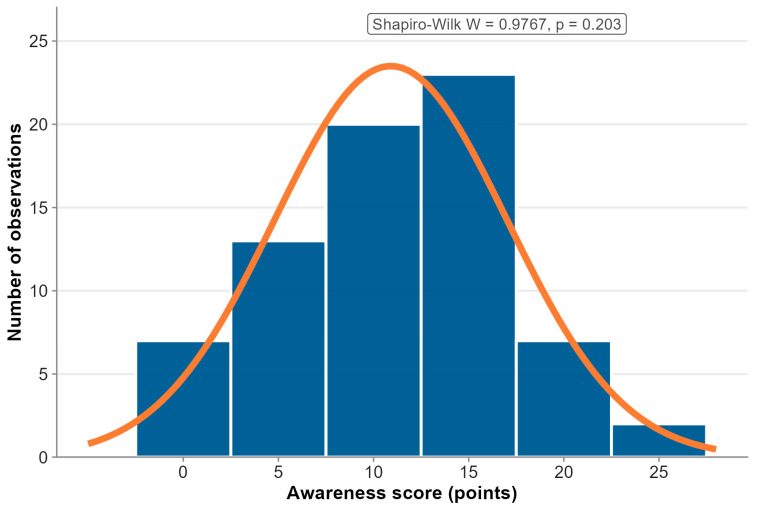
Distribution of the synthetic variable ‘awareness’. The orange line represents the fitted normal distribution curve.

A total of 18 questions were used to analyze patient awareness regarding dyspareunia, its causes, and available treatment options. Each question was scored 1–3 points depending on question type: yes/no questions (1 point for correct answer), multiple-choice questions (1 point per correct option or 3 points for “all of the above”), and open-ended questions (2 points for complete answers, 1 point for partial answers). Respondents were allowed a maximum of 24 points. The summed score constituted the synthetic variable ‘awareness’. Detailed scoring is presented in Supplementary Table S2. The distribution of the variable was normal (Shapiro–Wilk test *p* = 0.209) (Figure 1).

The internal consistency of the 18-item awareness scale was evaluated using Cronbach’s alpha. The raw alpha coefficient was 0.852, and the standardized alpha was 0.880, indicating good-to-excellent internal consistency and supporting the reliability of the aggregated awareness score as a unidimensional measure of knowledge.

**Figure 2:**
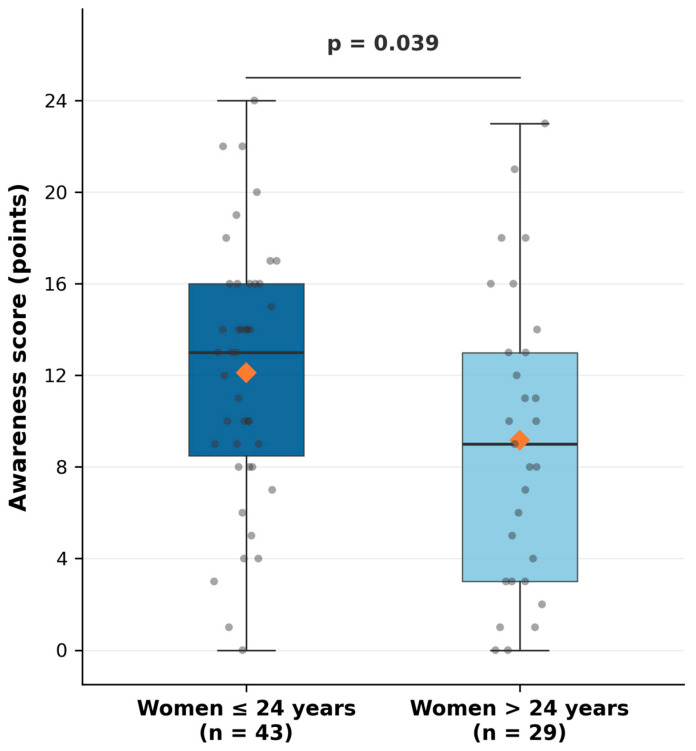
Comparison of mean awareness scores between age groups (≤24 years vs. >24 years). Boxes represent the interquartile range (Q1–Q3) with the median indicated by the horizontal line; whiskers extend to the minimum and maximum values. Grey dots represent individual participant scores; the orange diamond indicates the group mean.

Younger women (≤24 years) demonstrated significantly higher awareness scores (mean 12.1 ± 5.6) compared to older women (mean 9.1 ± 6.5; independent samples *t*-test, *p* = 0.039) (Table 2, Figure 2).

### 3.4. Item-Level Knowledge Analysis

Analysis of individual item scores revealed pronounced heterogeneity in the knowledge profile of the cohort (Table 3). Items addressing general pelvic floor anatomy and function (I1: 62.0% of maximum score), the biopsychosocial underpinnings of sexual dysfunction (I9: 67.1%), and the effect of pelvic floor muscles on personal sexual experience (I14: 72.2%) achieved the highest proportional scores. Awareness that pain during intercourse should prompt physiotherapy referral (I12: 66.7%) and that sneezing or coughing affects pelvic floor muscles (I2: 66.7%) were similarly relatively preserved.

**Figure 3:**
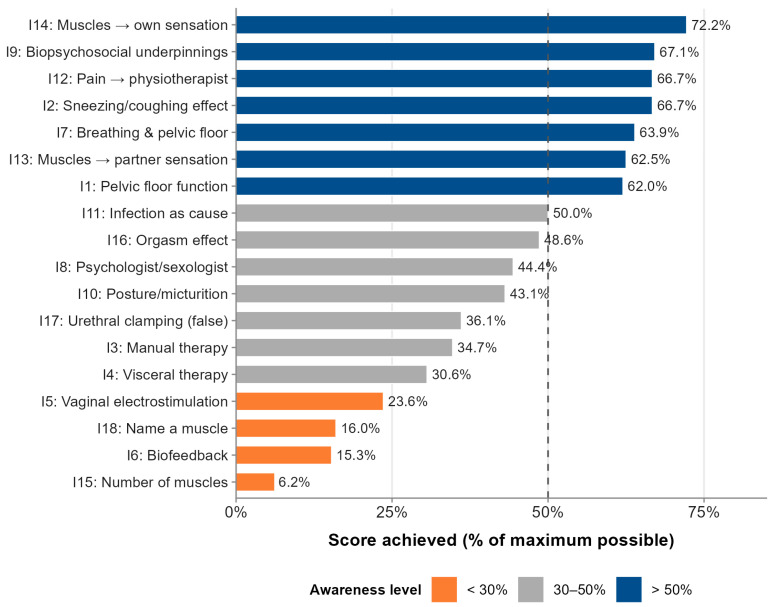
Proportional awareness scores for each of the 18 knowledge assessment items (% of maximum possible score). Items are ordered from lowest to highest. The dashed line marks the 50% threshold.

In contrast, knowledge gaps were most pronounced for items addressing specific physiotherapeutic modalities. Only 15.3% of participants correctly identified biofeedback as applicable in dyspareunia management (I6), and only 23.6% recognized vaginal electrostimulation as a component of pelvic floor therapy (I5). Visceral therapy techniques were correctly identified by 30.6% (I4), and manual therapy on tense pelvic muscles by 34.7% (I3). Knowledge of pelvic floor anatomy was particularly poor: only 6.2% of participants could correctly estimate the number of pelvic floor muscles (I15), and only 16.0% could name at least one constituent muscle (I18). The role of psychologist or sexologist consultation in dyspareunia treatment was correctly endorsed by 44.4% of participants (I8), and 36.1% correctly recognized that urethral clamping during urination is not an effective pelvic floor exercise (I17) (Figure 3).

### 3.5. Multiple Linear Regression Analysis of Awareness Predictors

To identify independent predictors of awareness, a multiple linear regression model was fitted with age, education level, pain duration, pain intensity (VAS), current physiotherapy use, and current treatment status as predictors. The overall model was statistically significant (F(6, 65) = 3.131, *p* = 0.009), explaining 22.4% of the variance in awareness scores (R^2^ = 0.224, adjusted R^2^ = 0.153). All variance inflation factors were below 1.66, indicating no multicollinearity (Table 4).

Age was a significant independent negative predictor of awareness (B = −0.188, β = −0.305, SE = 0.070, *p* = 0.009), indicating that each additional year of age was associated with a decrease of approximately 0.19 points on the awareness scale, independent of other variables. Current use of urogynecological physiotherapy was the strongest positive predictor (B = 6.791, β = 0.332, SE = 2.811, *p* = 0.019), with physiotherapy users scoring on average 6.8 points higher than non-users after adjustment for all other covariates. Education level, pain duration, pain intensity, and current treatment status did not independently predict awareness scores (all *p* > 0.18).

### 3.6. Subgroup Comparisons

Awareness scores were compared across subgroups defined by education level, pain duration, and physiotherapy use. Education level did not significantly affect awareness (Kruskal–Wallis H(2) = 2.976, *p* = 0.226), with mean scores of 7.1 ± 4.8, 11.3 ± 6.1, and 11.4 ± 6.2 for participants with primary, secondary, and tertiary education, respectively. Similarly, pain duration was not significantly associated with awareness (H(2) = 3.314, *p* = 0.191), with mean scores of 10.0 ± 5.6 for symptoms lasting less than 6 months, 13.1 ± 5.9 for 6 months to 2 years, and 10.2 ± 6.9 for more than 2 years.

All participants were classified as either current users (*n* = 7) or non-users (*n* = 65) of urogynecological physiotherapy; no participant reported past use without current continuation.

**Figure 4:**
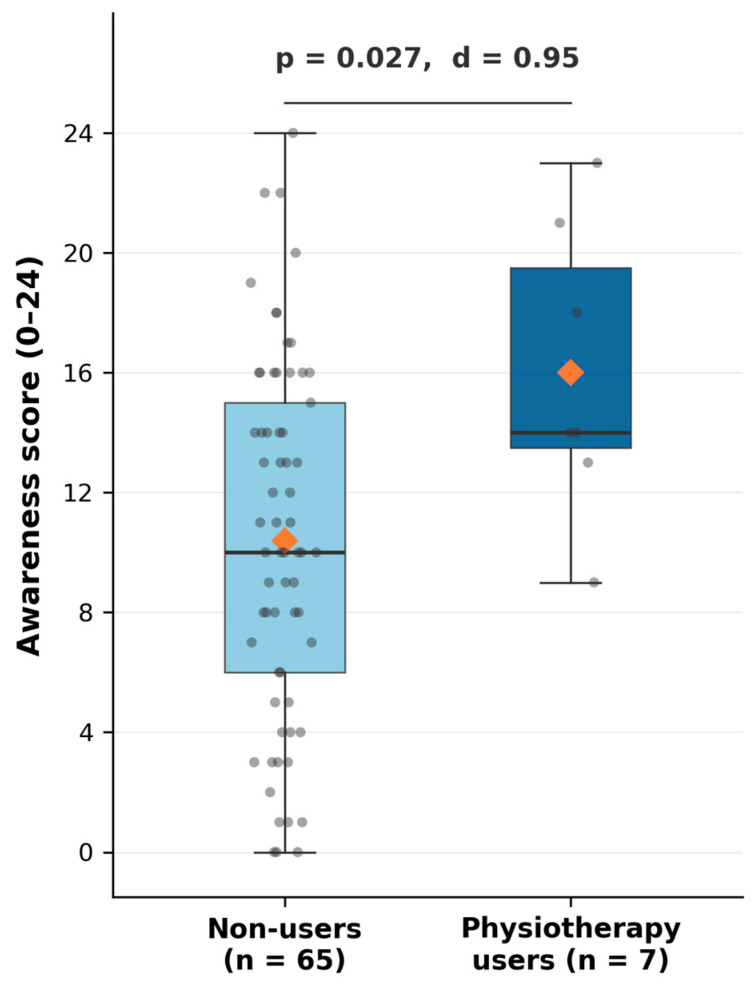
Awareness scores by urogynecological physiotherapy use. Boxes represent the interquartile range (Q1–Q3) with the median indicated by the horizontal line; whiskers extend to the minimum and maximum values. Grey dots represent individual participant scores; the orange diamond indicates the group mean. Mann–Whitney U test: U = 316.5, *p* = 0.027; rank-biserial r = 0.51.

Women currently using urogynecological physiotherapy demonstrated significantly higher awareness scores than those who had never accessed physiotherapy (16.0 ± 4.9 vs. 10.4 ± 6.0; Mann–Whitney U test U = 316.5, *p* = 0.027), with a large effect size (rank-biserial r = 0.51). Women aged ≤24 years scored significantly higher than older women (12.1 ± 5.6 vs. 9.1 ± 6.5; t(70) = 2.100, *p* = 0.039), with a medium effect size (Cohen’s d = 0.505) (Figure 4).

### 3.7. Correlational Analyses

Spearman’s rank correlations between awareness and continuous clinical variables revealed no statistically significant associations. Age showed a weak negative trend (rs = −0.203, *p* = 0.088), while pain intensity (rs = 0.139, *p* = 0.246), stress level (rs = 0.084, *p* = 0.484), and BMI (rs = −0.143, *p* = 0.232) were not correlated with awareness scores.

The weak and non-significant bivariate association between age and awareness should be interpreted in light of the multivariate regression results, where age emerged as a significant predictor after controlling for other covariates; this pattern is consistent with confounding or suppression effects in the bivariate analysis.

### 3.8. Association Between Dyspareunia Characteristics and Awareness

The dyspareunia characteristics—including positional dependency, penetration depth influence, partner-related factors, lubrication, sexual trauma history, pain timing, and level of sexual engagement—were evaluated in relation to awareness scores. None of these variables were significantly associated with awareness. A non-significant trend was observed for positional dyspareunia, with women reporting position-dependent pain scoring slightly higher on the awareness scale than those without positional dependency (11.9 ± 6.2 vs. 9.9 ± 5.9; Mann–Whitney U = 491.5, *p* = 0.082; Cohen’s d = 0.34). Awareness did not differ significantly by the most painful position (Kruskal–Wallis H = 0.980, *p* = 0.613), penetration depth influence (*p* = 0.613), partner consistency of pain (*p* = 0.687), lubrication influence (*p* = 0.951), lubricant use (*p* = 0.318), sexual trauma history (*p* = 0.431), timing of pain during intercourse (*p* = 0.891), or level of sexual engagement (*p* = 0.130).

## 4. Discussion

In this cross-sectional study of women with clinically confirmed dyspareunia, overall awareness of evidence-based, pelvic floor-focused and biopsychosocial interventions was modest. On a 24-point instrument with good internal consistency (standardized Cronbach’s α = 0.880), the mean awareness score was 10.9 ± 6.1. Multiple linear regression identified two independent predictors of awareness: younger age (β = −0.305, *p* = 0.009) and current use of urogynecological physiotherapy (β = 0.332, *p* = 0.019), together explaining 22.4% of the variance in awareness scores. Physiotherapy users scored on average 5.6 points higher than non-users—a large effect (rank-biserial r = 0.51)—suggesting that therapeutic contact may serve an educational function, although this finding should be interpreted with caution given the small number of current physiotherapy users (*n* = 7) and the correspondingly wide confidence interval (95% CI [1.177, 12.406]). Education level, pain duration, pain intensity, and current treatment status did not independently predict awareness. These findings underscore a persistent translational gap between research and lay knowledge and point to missed opportunities for early, noninvasive management.

Item-level analysis revealed a strikingly heterogeneous knowledge profile. Awareness was relatively preserved for general concepts such as the biopsychosocial underpinnings of sexual dysfunction (I9: 67.1%), pelvic floor function (I1: 62.0%), and the appropriateness of physiotherapy referral for dyspareunia (I12: 66.7%). In contrast, knowledge of specific physiotherapeutic modalities was critically low: biofeedback was correctly identified by only 15.3% of participants (I6), vaginal electrostimulation by 23.6% (I5), and visceral therapy techniques by 30.6% (I4). Anatomical knowledge was particularly deficient—only 6.2% could estimate the number of pelvic floor muscles (I15) and only 16.0% could name a constituent muscle (I18). This pattern suggests that women with dyspareunia may understand in general terms that physiotherapy is relevant to their condition, yet lack the specific knowledge needed to engage meaningfully with treatment components, evaluate treatment options, or advocate for appropriate referrals. These item-level gaps provide actionable targets for educational intervention design.

Our results align with the broader epidemiology and classification of sexual pain disorders. Population-based British data from Natsal-3 indicate that 7.5% of sexually active women report painful intercourse, with substantial associated decrements in sexual, relational, and mental health. This burden, coupled with under-recognition and the need for holistic assessment, makes public and clinician education critical [20]. At the nosological level, ICD-11 recognizes sexual pain-penetration disorder within conditions related to sexual health and emphasizes pain, involuntary pelvic floor contraction, and fear/anxiety components—elements that map to multimodal treatment needs [4]. A recent systematic review of biopsychosocial approaches for chronic pelvic pain further showed that guideline-recommended biopsychosocial treatments are supported by emerging RCT evidence, although heterogeneity in interventions and outcomes still limits implementation clarity [23].

The interventions with the strongest evidence for provoked vestibulodynia are precisely those with the lowest awareness in our sample. A multicenter randomized controlled trial demonstrated that multimodal PFPT—combining education, pelvic floor muscle exercises with biofeedback, manual therapy, dilation, and home exercises—reduced intercourse pain more than topical lidocaine, with benefits sustained at 6 months [11]. A systematic review and meta-analysis of 19 studies further supported physiotherapy-based interventions for dyspareunia, showing significant benefits for pain and quality of life, particularly in electrotherapy-based approaches; manual trigger point release was also identified as beneficial for pain reduction [24]. Similarly, a randomized clinical trial found CBCT superior to lidocaine across several pain-related, sexual, and psychological outcomes [12]. Contemporary expert consensus recommends psychological interventions and PFPT as first-line options in provoked vestibulodynia, reserving surgery for refractory cases [19].

Several low-awareness items in our instrument, including diaphragmatic breathing and relaxation-based strategies, are incorporated into some multimodal physiotherapy programs for dyspareunia. Foundational physiologic work has shown co-activation and functional coupling between the pelvic floor and abdominal wall musculature, particularly transversus abdominis, during respiratory and postural tasks [14]. Clinical programs integrating relaxation strategies, breathing, pain education, and pelvic floor relaxation/down-training have been associated with reductions in pain and dyspareunia severity across small trials and systematic review evidence, although the certainty of evidence remains limited and higher-quality randomized trials are still needed [24]. Addressing these mechanisms within PFPT and CBCT frameworks may help reframe dyspareunia as a treatable condition rather than an unavoidable “female problem,” a belief sometimes reflected in our respondents’ answers. Increasing awareness of these modalities, together with streamlined referral pathways, could therefore improve outcomes with low risk.

Dyspareunia encompasses several distinct phenotypes that often remain underrecognized by patients, each with specific implications for assessment and treatment. Postpartum dyspareunia is common and often transient, with higher rates reported after operative vaginal delivery or extensive perineal trauma [10,25,26]; patient education emphasizing that it is common, treatable, and frequently self-limiting may reduce distress and facilitate timely treatment-seeking. Adenomyosis warrants recognition as a distinct contributor to sexual pain, with higher symptom rates when coexisting with endometriosis and diminished response to surgical management in such cases [7,8]. Pelvic congestion syndrome, a frequently underdiagnosed vascular cause of chronic pelvic pain in which postcoital dyspareunia is a hallmark symptom, also merits consideration in the differential diagnosis [9]; its frequent co-occurrence with endometriosis reinforces the need to include vascular etiologies in educational materials. Together, these phenotypes illustrate the breadth of conditions underlying dyspareunia and highlight how limited patient awareness of this heterogeneity—reflected in the low knowledge scores observed in our cohort—may delay appropriate referral and management.

For endometriosis-related deep dyspareunia, both surgical and medical options are available. Laparoscopic excision can reduce pain and improve quality of life in appropriately selected patients, with the choice depending on disease severity, anatomic location, fertility goals, and patient preference [27,28]. For patients preferring medical management or with contraindications to surgery, oral GnRH antagonists (relugolix, elagolix) combined with add-back therapy provide meaningful symptom control across dysmenorrhea, non-menstrual pelvic pain, and dyspareunia, with longer-term data supporting maintained efficacy and acceptable safety [16,17,29]. However, awareness of both surgical and medical treatment pathways in our cohort was limited, with markedly low treatment utilization across modalities despite 15.3% of participants carrying a confirmed endometriosis diagnosis. This disconnect between available evidence-based options and patient knowledge underscores the need for clearer communication of the therapeutic landscape during clinical encounters.

The role of sexual position in modulating the frequency and severity of dyspareunia also deserves attention, although findings on positional and partner-related factors in our cohort should be interpreted as exploratory given the modest sample size and absence of correction for multiple testing. In our cohort, 55.6% of women reported that pain intensity varied depending on sexual position, with the knee-support (doggy style) position most frequently identified as the most painful (37.5%), a pattern broadly consistent with the findings of a recent prospective cohort study by Enzelsberger et al., in which 68.3% of endometriosis patients reported positional dependency, with the doggy style position being most frequently painful. The higher rate of positional dependency in the latter study likely reflects the specific endometriosis population, as compared to our heterogeneous dyspareunia cohort. In our sample, the majority of women (86.1%) did not report penetration depth as a pain-modifying factor, and 73.6% indicated that dyspareunia was not consistent across partners, which may tentatively suggest that relational and contextual factors contribute to pain experience—a hypothesis that warrants confirmation in adequately powered studies. Taken together with the evidence from Enzelsberger et al., these preliminary observations support further investigation of positional and partner-related factors as candidate dimensions for inclusion in clinical assessment tools and educational materials for women with sexual pain disorders [30].

In postmenopausal dyspareunia due to GSM, a recent systematic review concluded that vaginal estrogen, intravaginal prasterone, and oral ospemifene may improve vulvovaginal symptoms including dyspareunia in the short term, although long-term and comparative data remain limited [15]. By comparison, energy-based devices (e.g., fractional CO_2_ laser) have shown some benefit in sham-controlled trials, but the certainty of evidence remains low, warranting caution pending more definitive safety and efficacy data [31]. Patient education that prioritizes therapies with the best risk–benefit evidence is needed.

Our data also reveal uncertainty about interventions with limited supporting evidence, such as botulinum toxin A in provoked vestibulodynia [32] and off-label gabapentin for chronic pelvic pain without defined pathology [33], neither of which has shown consistent benefit over placebo in randomized trials. Educational materials should therefore not only promote evidence-based first-line options but also explain why some widely discussed interventions remain investigational and why stepped, multidisciplinary care is preferred.

Taken together, our findings emphasize the need for targeted educational initiatives for both the public and frontline clinicians (primary care, gynecology, urology, physiotherapy, psychology/sexology). Key messages include: dyspareunia is common and diagnosable; multimodal PFPT and psychological/sex therapy have some of the strongest supportive evidence in provoked vestibulodynia; condition-specific pharmacotherapy (e.g., GnRH antagonists for endometriosis, local hormonal therapy for GSM) should be prioritized over empiric neuropathic analgesics; and adjuncts such as breathing/relaxation and graded exposure are integral rather than “alternative.”

This study has several strengths. We focused on practical, modifiable knowledge domains and linked item content to contemporary evidence. The use of a clinical sample with confirmed dyspareunia enhances relevance, and the focus on pelvic floor therapy addresses a critical yet under-recognized treatment modality. Harmonizing outcomes with the recently developed Core Outcome Set for provoked vestibulodynia will also strengthen future trials and facilitate knowledge translation [34].

Several limitations should be considered when interpreting these findings. The sample size of 72 participants and single-country recruitment from a single tertiary gynecological clinic constrain both the statistical power to detect modest associations and the generalizability of results to broader populations. Women attending a specialist gynecological clinic in Poland may differ substantially from those in primary care, community, or non-European settings in terms of disease severity, healthcare-seeking behavior, cultural attitudes toward sexual health, and baseline awareness—and our findings should not be extrapolated to these populations without further validation. A formal count of women who declined participation was not recorded, precluding assessment of non-response bias. A particular limitation concerns the small number of current physiotherapy users (*n* = 7, 9.7%); the wide confidence interval for the regression coefficient (95% CI [1.177, 12.406]) reflects considerable uncertainty around this estimate, and replication in larger samples is needed before firm conclusions can be drawn. More broadly, the modest sample size limits the robustness of the multivariable regression model (R^2^ = 0.224, adjusted R^2^ = 0.153); although the two identified predictors reached statistical significance, the model’s explanatory power and the stability of coefficient estimates would benefit from replication in larger, adequately powered cohorts. The awareness instrument was developed specifically for this study and demonstrated good internal consistency (standardized Cronbach’s α = 0.880), supporting its reliability as a measure of knowledge in this domain; however, formal external validation has not yet been conducted and should be addressed in future work. The cross-sectional design captures associations rather than causal relationships between awareness and clinical outcomes. Furthermore, the number of statistical comparisons performed relative to the sample size increases the risk of both type I error (false positives) and type II error (false negatives, given limited power to detect small-to-moderate effects); as no correction for multiple testing was applied, positive findings from subgroup and exploratory analyses require confirmatory replication, while negative findings—including the absence of significant associations between dyspareunia characteristics and awareness—should not be interpreted as definitive evidence of no association. Additional factors including prior therapy exposure and digital health literacy may also influence awareness levels and warrant investigation in future studies.

The present study demonstrates the feasibility and potential value of systematically assessing patient awareness of evidence-based treatments for dyspareunia—a dimension that, to our knowledge, has not been formally evaluated in prior research. The identification of pronounced item-level knowledge gaps, particularly for specific physiotherapeutic modalities, provides a conceptual framework for designing targeted educational interventions. While the findings of this exploratory study should be interpreted within the constraints of a small, single-center sample, the approach itself—linking awareness assessment to clinical and sociodemographic predictors—offers a replicable model for future, larger-scale investigations.

Future work should validate a brief, reliable dyspareunia awareness instrument, co-design and test scalable educational interventions (print, web, and video formats) that explicitly address PFPT, CBCT/sex therapy, and condition-specific medical options, and evaluate their effects on care-seeking behavior, referral uptake, and patient-centered outcomes using validated measures (e.g., Female Sexual Function Index; Core Outcome Set for provoked vestibulodynia), with subgroup analyses by age and symptom phenotype. Such efforts could help close the evidence-to-practice gap highlighted by our cohort.

## 5. Conclusions

Women with dyspareunia demonstrate modest and heterogeneous awareness of evidence-based treatments. Knowledge deficits are most pronounced for specific physiotherapeutic modalities and basic pelvic floor anatomy, while general concepts such as the relevance of physiotherapy referral are relatively better understood. Younger age and active engagement with urogynecological physiotherapy were associated with higher awareness in this sample, suggesting that therapeutic contact may serve an educational function; however, the small number of physiotherapy users and the wide confidence interval for this estimate warrant cautious interpretation and replication in larger cohorts.

These findings highlight a substantial gap between the availability of evidence-based treatments and patient knowledge of these options. Targeted educational interventions addressing specific knowledge deficits are needed across primary care, gynecology, and physiotherapy settings. Streamlined referral pathways to pelvic floor physiotherapy may simultaneously improve treatment access and patient awareness. Future studies should validate a dedicated dyspareunia knowledge instrument, recruit larger and more diverse samples, and evaluate the effect of educational programs on treatment-seeking behavior and clinical outcomes.

More broadly, this study establishes a conceptual and methodological framework for assessing patient awareness of dyspareunia treatments, which may be adapted and validated in future multi-center studies with larger and more diverse populations.

## Figures and Tables

**Table 1:** Sociodemographic and clinical characteristics of the study population (*N* = 72).

Variable	*n* (%) or Mean ± SD
Sociodemographic characteristics	
Age (years), mean ± SD	27.7 ± 9.9
Age (years), median (range)	23.5 (17–54)
≤24 years	43 (59.7%)
>24 years	29 (40.3%)
Education	
Primary	7 (9.7%)
Secondary	31 (43.1%)
Tertiary	34 (47.2%)
Place of residence	
Large city (>100,000)	38 (52.8%)
Medium town (20,000–100,000)	13 (18.1%)
Small town (<20,000)	6 (8.3%)
Rural area	15 (20.8%)
Marital status	
Single	50 (69.4%)
Married	22 (30.6%)
BMI (kg/m^2^), mean ± SD	24.0 ± 5.0
Reproductive history	
Ever pregnant	22 (30.6%)
Nulliparous	52 (72.2%)
Mode of delivery (among parous, *n* = 20)	
Vaginal	14 (70.0%)
Caesarean section	6 (30.0%)
Dyspareunia characteristics	
Duration of dyspareunia	
<6 months	34 (47.2%)
6 months–2 years	20 (27.8%)
>2 years	18 (25.0%)
Pain intensity (VAS 0–10), mean ± SD	4.4 ± 2.0
Pain intensity (VAS 0–10), median (range)	4.0 (0–10)
Attributed onset of dyspareunia	
Genital/urinary infection	24 (33.3%)
Since sexual debut	18 (25.0%)
After pregnancy/childbirth	17 (23.6%)
Psychological factors	13 (18.1%)
Pain type	
Stabbing	50 (69.4%)
Burning	22 (30.6%)
Pain location	
Superficial	29 (40.3%)
Deep vaginal	25 (34.7%)
Diffuse	10 (13.9%)
Urethral area	8 (11.1%)
Sexual activity frequency	
Several times/week	20 (27.8%)
Once/week	14 (19.4%)
1–2 times/month	29 (40.3%)
Not sexually active	9 (12.5%)
Age at first intercourse (years), mean ± SD	18.4 ± 2.2
Gynecologic and medical history	
History of genital infections	47 (65.3%)
Dysmenorrhea	52 (72.2%)
Back pain	50 (69.4%)
Diagnosed gynecologic conditions	
None	19 (26.4%)
Genital infections	22 (30.6%)
Cervical erosion	15 (20.8%)
Endometriosis	11 (15.3%)
Ovarian cysts	5 (6.9%)
Current hormonal contraception	25 (34.7%)
Ever used hormonal contraception	48 (66.7%)
History of sexual trauma	21 (29.2%)
Treatment and healthcare utilization	
Currently treated for dyspareunia	9 (12.5%)
Current urogynecological physiotherapy	7 (9.7%)
Specialist consultation for dyspareunia	
None	55 (76.4%)
Gynecologist	14 (19.4%)
Psychologist/sexologist	3 (4.2%)
Discussed dyspareunia with gynecologist	15 (20.8%)
Discussed dyspareunia with partner	56 (77.8%)
Ever attempted pelvic floor exercises	29 (40.3%)
Current pelvic floor exercise frequency	
None	53 (73.6%)
≤2/week to 1/month	13 (18.1%)
≥3/week	6 (8.3%)
Psychological and relational factors	
Stress level (1–5), mean ± SD	3.5 ± 1.0
Level of sexual engagement	
Fully	17 (23.6%)
Usually	40 (55.6%)
Not always	15 (20.8%)
Emotional response to sexual activity	
Very positive	40 (55.6%)
Neutral	24 (33.3%)
Unable to determine	8 (11.1%)

**Table 2:** Awareness scores for total cohort and by age subgroup (≤24 years vs. >24 years).

Awareness			
	General Population	Women ≤ 24 Years	Women > 24 Years
Mean ± SD	10.9 ± 6.1	12.1 ± 5.6	9.1 ± 6.5
Median	11	13	9
Minimum	0	0	0
Maximum	24	24	23

**Table 3:** Item-level awareness scores (*N* = 72).

Item	Description	Mean Score	Maximum	% of Maximum
I1	Pelvic floor function	1.86	3	62.0%
I2	Sneezing/coughing effect	0.67	1	66.7%
I3	Manual therapy on tense muscles	0.35	1	34.7%
I4	Visceral therapy	0.31	1	30.6%
I5	Vaginal electrostimulation	0.24	1	23.6%
I6	Biofeedback	0.15	1	15.3%
I7	Breathing and pelvic floor	0.64	1	63.9%
I8	Psychologist/sexologist consultation	0.44	1	44.4%
I9	Biopsychosocial underpinnings	2.01	3	67.1%
I10	Posture/micturition as prevention	0.43	1	43.1%
I11	Infection as cause of dyspareunia	0.50	1	50.0%
I12	Pain as reason to see physiotherapist	0.67	1	66.7%
I13	Muscles and partner sensation	0.63	1	62.5%
I14	Muscles and own sensation	0.72	1	72.2%
I15	Number of pelvic floor muscles	0.12	2	6.2%
I16	Effect of orgasm	0.49	1	48.6%
I17	Urethral clamping (false belief)	0.36	1	36.1%
I18	Naming a pelvic floor muscle	0.32	2	16.0%

Note: Items I1 and I9 allowed multiple correct responses (1 point each) or “All of the above” (3 points). Items I15 and I18 were open-ended and scored 0–2 points. All other items were binary (correct = 1, incorrect/don’t know = 0). Total maximum awareness score = 24 points.

**Table 4:** Multiple linear regression: predictors of awareness score (*N* = 72).

Predictor	B	SE	β	t	*p*	95% CI	VIF
Age	−0.188	0.070	−0.305	−2.702	0.009 **	[−0.327, −0.049]	1.068
Education level	1.333	1.082	0.144	1.231	0.223	[−0.829, 3.495]	1.143
Pain duration	0.453	0.897	0.061	0.505	0.615	[−1.338, 2.244]	1.231
VAS pain intensity	0.493	0.365	0.159	1.352	0.181	[−0.235, 1.221]	1.161
Physiotherapy use	6.791	2.811	0.332	2.416	0.019 *	[1.177, 12.406]	1.578
Current treatment	−1.684	2.577	−0.092	−0.654	0.516	[−6.831, 3.463]	1.652

Note: B = unstandardized regression coefficient; SE = standard error; β = standardized coefficient; t = t-statistic; 95% CI = 95% confidence interval for B. Overall model: R^2^ = 0.224, adjusted R^2^ = 0.153, F(6, 65) = 3.131, *p* = 0.009. All VIF values < 1.66 (no multicollinearity). * *p* < 0.05; ** *p* < 0.01.

## Data Availability

The original contributions presented in this study are included in the article/Supplementary Material. Further inquiries can be directed to the corresponding author.

## Supplementary Materials

The following supporting information can be downloaded at: https://www.mdpi.com/article/10.3390/jcm15093408/s1, the survey instrument and scoring methodology are provided as Supplementary Tables S1 and S2. Table S1: Complete survey instrument assessing patient characteristics, dyspareunia symptoms, and awareness of pelvic floor therapy. The 82-item questionnaire comprised nine sections: (A) sociodemographic data, (B) dyspareunia characteristics, (C) menstrual characteristics, (D) reproductive history, (E) contraception and gynecologic history, (F) medical history, (G) psychological and relational factors, (H) treatment history, and (I) knowledge assessment; Table S2: Scoring system for knowledge assessment items (Section I of survey instrument). Points allocated for each response option, with maximum possible score indicated for each item. Total maximum score: 24 points.
