# Supplementary material for: Awareness of Evidence-Based Treatments Among Women with Dyspareunia: A Cross-Sectional Survey Study
Source: J Clin Med. 2026 Apr 29;15(9):3408. doi: 10.3390/jcm15093408 (PMC13163913; doi:10.3390/jcm15093408)
Supplement: Supplementary file 1 [file jcm-15-03408-s001.zip › jcm-4238824-supplementary.pdf]

Supplementary Table S1. Complete survey instrument assessing patient characteristics, dyspareunia symptoms, and awareness of pelvic floor therapy. The 82-item questionnaire comprised nine sections: (A) sociodemographic data, (B) dyspareunia characteristics, (C) menstrual characteristics, (D) reproductive history, (E) contraception and gynecologic history, (F) medical history, (G) psychological and relational factors, (H) treatment history, and (I) knowledge assessment.

No.	Question Item	Response Options
**SECTION A: SOCIODEMOGRAPHIC DATA**		
A1	Please indicate your age	[Open response - years]
A2	What is your educational background	Primary / Lower secondary / Vocational / Secondary / Tertiary
A3	Place of residence	Rural / Small town (up to 20,000 inhabitants) / Medium town (20,000 to 100,000 inhabitants) / Large town (more than 100,000 inhabitants)
A4	What is your marital status	Single / Married / Divorced / Widowed
A5	Please indicate your height	[Open response - cm]
A6	Please indicate your weight	[Open response - kg]
**SECTION B: DYSPAREUNIA CHARACTERISTICS**		
B1	How long have you suffered from pain during intercourse	Since 3 months / Since 6 months / Since 1 year / Since 2 years / Longer than 2 years
B2	When did you first experience pain during intercourse	Right after starting intercourse / During pregnancy / After pregnancy / During menopause / After menopause / After infection of genital tract and/or urinary tract / Difficult to say / Other
B3	At what age did you have your first sexual intercourse	[Open response - years]
B4	How often do you have intercourse	Several times a week / Once a week / Less than once a week / Once a month / Less than once a month / No intercourse
B5	On a scale from 0 to 10, how would you rate the intensity of pain during intercourse (0 = no pain, 10 = most severe pain)	[VAS scale 0-10]
B6	What is the nature of pain during intercourse	Burning / Stabbing / Sharp / Other (specify)

No.	Question Item	Response Options
B7	In what area do you experience pain during intercourse (multiple answers possible)	Vaginal entrance / Deep inside the vagina / Urethral area / Anal area / Area between vaginal entrance and anus / Other (specify)
B8	In which situation do you feel pain during intercourse (multiple answers possible)	At the entrance to the vagina / At deep penetration / At the beginning of intercourse, after some time the pain disappears / Pain appears during intercourse / Pain appears at the end of intercourse / Pain appears after the end of intercourse / Other
B9	Do you experience pain every time you have intercourse	Yes / No / Difficult to say
B10	Does your partner's anatomy (penis size) affect pain during intercourse	Yes / No / I don't know
B11	Have you experienced pain during intercourse with all your partners	Yes / No / Difficult to say
B12	Is pain caused by a particular position during intercourse	Yes / No / Difficult to say
B13	If pain is caused by a specific position, in which positions do you feel pain	In the "missionary" position (partner on top) / In the side position (lying sideways) / In the "riding position" (woman on top) / In the position from behind (supported kneeling, partner on back) / Not applicable / In any other position (specify)
B14	Does vaginal lubrication affect the pain you feel during intercourse	Yes / No / I don't know
B15	Do you use lubricants (lube, natural oils, saliva, etc.) during intercourse	Yes / No / I sometimes use
**SECTION C: MENSTRUAL CHARACTERISTICS**		
C1	Have you noticed a dependence of your pains on	Yes / No / I don't know

No.	Question Item	Response Options
	the day of your menstrual cycle	
C2	In relation to your menstrual cycle, when does the pain occur	Before menstruation / During menstruation / After menstruation / Before ovulation / During ovulation / After ovulation / Not applicable
C3	Do you suffer from painful periods	Yes / No / Don't know
C4	On a scale from 0 to 10, how would you describe the pain during menstruation (0 = no pain, 10 = most painful)	[VAS scale 0-10]
C5	How would you describe the location of the pain during menstruation	Pain in the lower abdomen / Pain spreading to the whole abdomen / Pain radiating to the back / Pain radiating to the thighs / Other pain (specify)
C6	Are you accompanied by any other complaints during menstruation	Nausea / Headache / Dizziness / Diarrhoea / Other complaints (specify)
**SECTION D: REPRODUCTIVE HISTORY**		
D1	Were you pregnant	Yes / No
D2	Have you had multiple pregnancies	Yes / No / Not applicable
D3	Do you have children, if yes, how many	One / Two / Three / More than 3 / I have no children
D4	How did the birth(s) take place (multiple answers possible)	By natural childbirth / Cesarean section / Not applicable
D5	Please indicate if you experienced any of the following problems during your pregnancy	Upper respiratory infections, colds / Intimate and/or bladder infections / Hypertension / Gestational diabetes / Cholestasis / Fetal distress / Pre-eclampsia / Cervical insufficiency / Carriage of pregnancy / Severe anaemia / Placental insufficiency and risk of fetal hypoxia / Pregnancy without any complications / Other (specify) / Not applicable

No.	Question Item	Response Options
D6	Were there any complications during delivery	Yes / No / Not applicable
D7	Please indicate which perinatal complications occurred (multiple answers possible)	Perineal incision / Perineal rupture / Cupping of the uterine cavity / Rupture of the labia / Separation of sutures in the perineum / Haematoma in the postpartum wound / Infections in the vaginal area, vulva in the puerperium / Other complications (specify) / Not applicable
D8	Have you received scar therapy following delivery by caesarean section (multiple answers possible)	Yes / No / Not applicable
D9	Did you experience any complications with your scar after the caesarean section	Pain in the scar region / Pulling sensation in the scar region / Lack of sensation in the scar region / Palsy in the scar region / Other (specify) / Not applicable
D10	Please tick other activities during which you feel pain in the perineum	Cycling / Inserting a tampon / Gynaecological examination / Inserting vaginal medicines / Inserting a menstrual cup / No pain in other activities / Other (specify)
**SECTION E: CONTRACEPTION AND GYNECOLOGIC HISTORY**		
E1	Are you taking hormonal contraception	Yes / No
E2	Have you ever taken hormonal contraception	Yes / No
E3	Do you have infections or inflammations of your genitals	Yes / No / I don't know
E4	How often do you get intimate infections	Once a month / Once a quarter / Once every six months / Once a year / Less than once a year / I do not suffer from intimate infections
E5	Please tick the genital diseases that you have been diagnosed with	Erosion of the cervical region / Ovarian cysts / Uterine myomas / Neoplastic conditions of the reproductive organs /

No.	Question Item	Response Options
		Endometriosis / Polycystic ovary syndrome / Bacterial infection of the vagina / Fungal infection of the vagina / Viral infection of the vagina / Other (specify) / No diagnosis of genital diseases
E6	Are you suffering from endometriosis	Yes / No / In the process of diagnosis / Don't know
E7	Have you ever undergone genital surgery	Yes (specify operation) / No
E8	Have you ever had any pelvic surgery	Yes (specify operation) / No
E9	Have you ever had any genital anomalies	Yes (specify) / No
**SECTION F: MEDICAL HISTORY**		
F1	Please mark the chronic diseases which you were diagnosed with	Hypertension / Heart failure / Systemic lupus erythematosus / Diabetes mellitus / Hashimoto's / Polycystic ovary syndrome / Other (specify) / No chronic diseases
F2	Do you suffer from back pain	Yes / No
F3	On a scale from 0 to 10, to what extent do you describe your back pain (0 = no pain, 10 = most severe pain)	[VAS scale 0-10]
F4	Please tick the part of your spine that hurts	Sacral / Lumbar / Thoracic / Cervical / Not applicable
**SECTION G: PSYCHOLOGICAL AND RELATIONAL FACTORS**		
G1	To what extent do you describe the level of stress in your life (0 = no stress, 5 = high levels of stress)	[Scale 0-5]
G2	Have you ever had a traumatic experience related to your sex life	Yes / No / I don't know
G3	Please mark on a scale of 0 to 5 what emotions does the	[Scale 0-5]

No.	Question Item	Response Options
	memory of your first sexual intercourse evoke in you (0 = very negative emotions, 5 = very positive emotions, very good memories)	
G4	What emotions do you feel about cohabitation	Very good emotions / I feel no strong emotions (neutral attitude) / Bad emotions / I cannot say
G5	How involved are you during intercourse	I am fully involved / I am usually involved / I am not always involved / I do not feel the need to have intercourse
**SECTION H: TREATMENT HISTORY**		
H1	Do you treat or have you treated complaints related to sexual intercourse	Yes / No
H2	Which complaints related to sexual intercourse have you treated	Dyspareunia / Vaginosis / Vulvodynia / Other (specify) / Not applicable
H3	How often do you receive urogynaecological physiotherapy	Several times a week / Twice a week / Once a week / Once every 2 weeks / Once a month / Less than once a month / I don't use it
H4	Please indicate for what reason do you use urogynaecological physiotherapy (multiple answers possible)	Dyspareunia / Vaginismus / Endometriosis / Painful menstrual periods / Back pain / Diastasis recti abdominis / Urinary incontinence / Fecal incontinence / Scars from pelvic surgery / Preparation for pregnancy / Preparation for delivery during pregnancy / Other reason / I do not use physiotherapy
H5	Do you use any specialist because of pain problems during intercourse (multiple answers possible)	Psychologist / Psychotherapist / Sexologist / Psychiatrist / Gynaecologist / Any other specialist (specify)
H6	Have you talked to a gynaecologist about your own problems with painful intercourse	Yes / No
H7	Have you talked to your partner about your own	Yes / No

No.	Question Item	Response Options
	problems with painful intercourse	
H8	Have you ever exercised your pelvic floor muscles	Yes / No / I don't know
H9	How often do you do pelvic floor muscle training	Every day / 3-4 times a week / 1-2 times a week / Once a month / Less than once a month
**SECTION I: KNOWLEDGE ASSESSMENT**		
**I1**	What is the function of the pelvic floor	Support of the abdominal organs / Stabilisation of the spine / Proper functioning of the sphincters / All of the above / Don't know
**I2**	Does how you sneeze or cough affect your pelvic floor muscles	Yes / No / I don't know
**I3**	Does pelvic floor muscle therapy use manual therapy techniques on 'tense' pelvic floor muscles	Yes / No / Don't know
**I4**	Can visceral therapy techniques (abdominal shell therapy, instrument work) be used in pelvic floor muscle therapy	Yes / No / Don't know
**I5**	Can vaginal electrostimulation be used in pelvic floor muscle therapy	Yes / No / Don't know
**I6**	Can biofeedback be used in pelvic floor therapy for dyspareunia	Yes / No / I don't know
**I7**	Does learning to breathe properly affect the pelvic floor muscles	Yes / No / I don't know
**I8**	Should consultation with a psychologist or sexologist be recommended during dyspareunia therapy	Yes / No / I don't know

No.	Question Item	Response Options
**I9**	Sexual dysfunction in women underlies dysfunction	Somatic / Mental / Social / All of the above
**I10**	Is an appropriate position for micturition and defecation considered preventive in pelvic floor muscle dysfunction	Yes / No / Don't know
**I11**	Can painful intercourse be related to having passed a urinary tract infection in the past	Yes / No / I don't know
**I12**	Should pain during intercourse be a reason to see a physiotherapist	Yes / No / It is normal / I don't know
**I13**	Can your fit pelvic floor muscles increase your partner's sensation during intercourse	Yes / No / I don't know
**I14**	Can fit pelvic floor muscles increase your own sexual experience	Yes / No / I don't know
**I15**	How many pelvic floor muscles are there	4 / 6 / 8 / 9 / 11 / Don't know
**I16**	Is exercising the pelvic floor muscles during urination, by clamping the urethra, an effective exercise	Yes / No / I don't know
**I17**	What effect does orgasm have on the pelvic floor muscles	Positive / Neutral / Negative
**I18**	Can you name at least one muscle that makes up the pelvic floor	[Open response]

Note: Section I items (I1-I18) were scored to create an aggregate "awareness" variable ranging from 0-24 points. Detailed scoring methodology for each item is presented in Table 2. VAS = Visual Analogue Scale.

Supplementary Table S2. Scoring system for knowledge assessment items (Section I of survey instrument). Points allocated for each response option, with maximum possible score indicated for each item. Total maximum score: 24 points.

No.	Question	Response Options	Points	Max
1	What is the function of the pelvic floor?			3
		Supporting the abdominal organs	1	
		Stabilisation of the spine	1	
		Proper functioning of the sphincters	1	
		All of the above	3	
		Do not know	0	
2	Does sneezing or coughing affect your pelvic floor muscles?			1
		Yes	1	
		No / I don't know	0	
3	Does pelvic floor muscle therapy use manual therapy techniques on tense pelvic muscles?			1
		Yes	1	
		No / I don't know	0	
4	Can visceral therapy techniques (abdominal shell therapy) be used in pelvic floor muscle therapy?			1
		Yes	1	
		No / I don't know	0	
5	Can vaginal electrostimulation be used in pelvic floor muscle therapy?			1
		Yes	1	
		No / I don't know	0	
6	Can biofeedback be used during pelvic floor therapy for dyspareunia?			1
		Yes	1	
		No / I don't know	0	
7	Does learning to breathe properly affect the pelvic floor muscles?			1
		Yes	1	
		No / I don't know	0	
8	Should consultation with a psychologist or sexologist be recommended when treating dyspareunia?			1
		Yes	1	
		No / I don't know	0	

No.	Question	Response Options	Points	Max
9	Female sexual dysfunction underlies dysfunction:			3
		Somatic	1	
		Mental	1	
		Social	1	
		All of the above	3	
		Do not know	0	
10	Is the correct position of micturition and defecation considered preventative in pelvic floor muscle dysfunction?			1
		Yes	1	
		No / I don't know	0	
11	Could painful intercourse be related to having passed a urinary tract infection in the past?			1
		Yes	1	
		No / I don't know	0	
12	Should pain during intercourse be a reason to see a physiotherapist?			1
		Yes	1	
		No, it is normal / I don't know	0	
13	Can your fit pelvic floor muscles increase your partner's sensation during intercourse?			1
		Yes	1	
		No / I don't know	0	
14	Can fit pelvic floor muscles enhance your own sexual experience?			1
		Yes	1	
		No / I don't know	0	
15	How many pelvic floor muscles are there?			2
		Correct answer (11 muscles)	2	
		Approximate number	1	
		No answer / Don't know	0	
16	What effect does orgasm have on the pelvic floor muscles?			1
		Positive	1	
		Negative / Neutral / I don't know	0	

No.	Question	Response Options	Points	Max
17	Is exercising the pelvic floor muscles during urination by clamping the urethra an effective exercise?			1
		No / I don't know	1	
		Yes	0	
18	Can you name at least one muscle that makes up the pelvic floor muscles?			2
		Full name of at least one muscle	2	
		Partial name or muscle group	1	
		No answer / Don't know	0	
			TOTAL	24

Note: Items 1 and 9 allowed multiple individual correct responses (1 point each) or selection of "All of the above" (3 points). Items 15 and 18 were open-ended questions scored based on response completeness (0-2 points). All other items were binary (correct = 1 point, incorrect/don't know = 0 points). The aggregate awareness score ranged from 0 to 24 points.
